# Cold adaptation strategies in plants—An emerging role of epigenetics and antifreeze proteins to engineer cold resilient plants

**DOI:** 10.3389/fgene.2022.909007

**Published:** 2022-08-25

**Authors:** Satyakam, Gaurav Zinta, Rajesh Kumar Singh, Rajiv Kumar

**Affiliations:** ^1^ Biotechnology Division, CSIR-Institute of Himalayan Bioresource Technology, Palampur, Himachal Pradesh, India; ^2^ Academy of Scientific and Innovative Research (AcSIR), Ghaziabad, India

**Keywords:** cold acclimation, freezing stress, DNA methylation, genetic engineering, antifreeze proteins

## Abstract

Cold stress adversely affects plant growth, development, and yield. Also, the spatial and geographical distribution of plant species is influenced by low temperatures. Cold stress includes chilling and/or freezing temperatures, which trigger entirely different plant responses. Freezing tolerance is acquired via the cold acclimation process, which involves prior exposure to non-lethal low temperatures followed by profound alterations in cell membrane rigidity, transcriptome, compatible solutes, pigments and cold-responsive proteins such as antifreeze proteins. Moreover, epigenetic mechanisms such as DNA methylation, histone modifications, chromatin dynamics and small non-coding RNAs play a crucial role in cold stress adaptation. Here, we provide a recent update on cold-induced signaling and regulatory mechanisms. Emphasis is given to the role of epigenetic mechanisms and antifreeze proteins in imparting cold stress tolerance in plants. Lastly, we discuss genetic manipulation strategies to improve cold tolerance and develop cold-resistant plants.

## Introduction

Plants are sessile organisms constantly challenged by environmental stresses such as temperature extremes, UV radiation, salinity, drought, flooding, mineral toxicity, and pathogen attack. Among different environmental stresses, cold severely alters membrane fluidity, water and ionic balance, generates reactive oxygen species (ROS) that impair DNA, RNA, and protein stability, hamper photosynthetic efficiency and slow down biochemical reactions. These cellular and physiological changes reduce growth, development, and productivity and limit the geographical distribution of plants (Steponkus and Lynch, 1989; Hüner et al., 2013; Barrero-Sicilia et al., 2017; Bailey-Serres et al., 2019). Therefore, understanding the plant responses and adaptation processes is important for developing of cold resilient plants, which is critical for global food security. The last decades have witnessed tremendous efforts to understand cold adaptation mechanisms in plants (Theocharis et al., 2012; Jeon & Kim, 2013; Zhao et al., 2015; Shi et al., 2018; Guo et al., 2018; Baier et al., 2019; Ding et at., 2019).

Plants encounter two types of low-temperature regimes in their natural habitat. The temperature range between 0 and 15°C causes cold/chilling stress, while temperatures below 0°C cause freezing stress, and distinct adaptive mechanisms help plants to deal with these two cold stress types. Plants use avoidance and tolerance strategies to mitigate cold stress. Avoiding mechanism involves preventing the formation of ice crystals inside the cell and is primarily associated with structural aspects. However, cold tolerance involves acquiring tolerance to low non-freezing temperature through a process known as cold acclimation, which includes prior exposure to nonlethal low temperature (Guy 1990; Thomashow, 1999). Cold acclimation is mainly characterized by the regulation of gene expression and metabolic changes that lead to various morphological, biochemical, and physiological alterations in plants (Liu et al., 2018; Liu et al., 2019; Wang B. et al., 2020; Fang et al., 2021; Guo et al., 2021).

Cold acclimation involves plasma membrane rigidification that affects ion concentration and metabolite transport from apoplast, endomembranes, and organelles, which initiates downstream cold signaling. These signaling cascades ultimately regulate the expression of cold-responsive (COR) genes. COR genes are induced by C-repeat Binding Factors (CBFs), which are under the control of the Inducer of CBF Expression (ICE). CBFs are genes encoding transcriptional activators having important roles in plant cold adaptation. Further, the ICE-CBF-COR regulatory module is a central pathway affecting cold response in plants (Chinnusamy et al., 2003; Jin et al., 2018). Cold inducible genes regulate the synthesis of compatible solutes (soluble sugars and proline), pigments (xanthophylls and carotenoids), and cold-responsive proteins like antifreeze proteins (AFPs), late embryogenesis abundant (LEA) proteins, heat shock proteins (HSPs), cold shock proteins (CSPs), and dehydrins, which eventually impart cold tolerance (Griffith et al., 1992a; Rinehart et al., 2007; Latowski et al., 2011; Liu et al., 2016; Yu et al., 2017; Meena et al., 2019). AFPs provide cold tolerance by arresting the growth of intracellular ice formation by binding to miniature ice-crystals formed due to freezing stress. Cold stress driven regulation of gene expression often depends on chromatin properties and small RNAs. In recent years, it has been documented that dynamics of histone modifications, DNA methylation, and biogenesis of miRNAs termed as “epigenetic regulators” were largely involved in the regulation of transcriptional and post-transcriptional gene expression in response to abiotic stress, including cold (Park et al., 2018; Hereme et al., 2021). Most of the epigenetic modifications are stable in the genome and forwarded to the next generation as epigenetic stress memory that could act more effectively towards subsequent cold stress. Our understanding of key genes that impart cold/freezing tolerance is crucial for developing cold resilient plants. The key genes primarily include signaling components like protein kinases, ion transporter, biochemical/ metabolic enzymes, and transcription factors are potential targets of crop improvement. With the help of genetic engineering techniques, the generation of overexpression/silencing line of key regulatory genes involved in cold adaptation could be an important strategy for developing cold resilient plants (Wang Q. et al., 2020; Li et al., 2021; Rivero et al., 2022).

This review summarises various aspects of cold stress response in plants and discusses the underlying adaptive mechanisms. Specifically, we discuss the role of epigenetic mechanisms and antifreeze proteins in cold stress tolerance. At the end, we highlight how modern genetic engineering tools can be utilized to develop cold resilient crops, and the industrial application of antifreeze proteins is also discussed. This review should provide us to characterize the process responsible for cold tolerance in plants that will be helpful in developing stress-resilient crops.

## Cold sensing and signaling pathways

**FIGURE 1:**
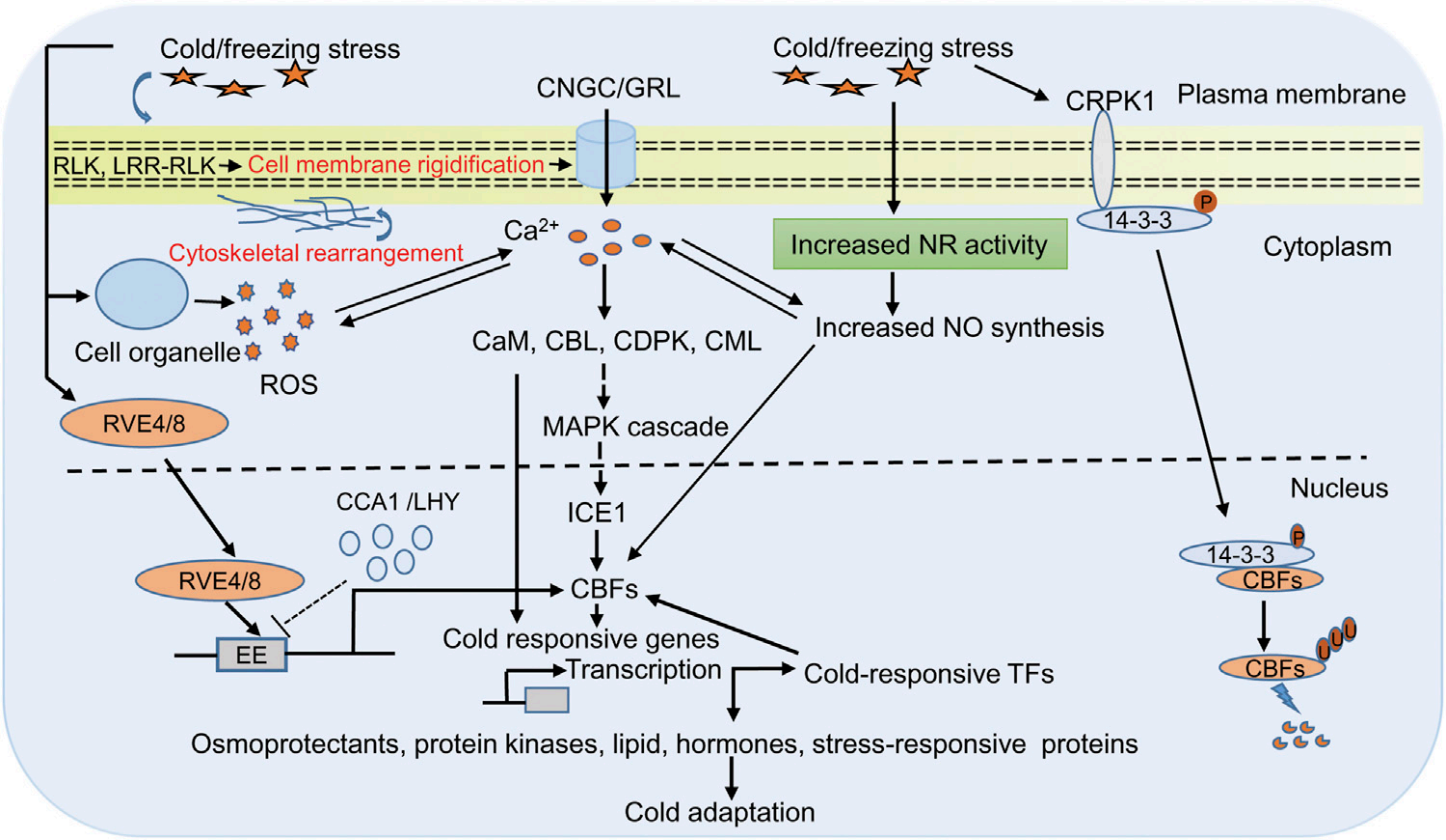
Representative diagram of cold responsive signaling pathway in plants. Plants sense cold/freezing signals through membrane receptor (RLK and LRR-RLK) and membrane rigidification. Cold sensing activates calcium channels (CNGC/GRL) that lead to increase Ca^2+^ in cytoplasm, which in turn activates of Ca^2+^ related protein likases (CaM, CML, CDPKs, and CBLs) and downstream signaling including MAPK signaling. These signaling cascades finally interacts with ICE1 and controls expression of CBFs/COR genes. COR genes encode proteins required for the biosynthesis of osmoprotectants, cryoprotectants, protein kinases, lipid, hormone, and stress-responsive proteins that are directly involved in cold tolerance. In addition, COR gene-dependent responses involve expression of diverse cold-induced transcription factors, which regulates CBFs expression in either positive or negative manner. The cold/freezing stress and increased Ca^2+^ activates the NADPH to generate more ROS. ROS and Ca^2+^ regulate each other’s concentration, and this cross talk controls the expression of defense gene in the nucleus. Cold/freezing stress also triggers NO synthesis that is essential for cold acclimation response through CBF dependent manner. In another cold signaling pathway, 14-3-3 protein get phosphorylation by CRPK1 followed by translocation from the cytoplasm to the nucleus where it interacts with CBFs and trigger its degradation through the 26S proteasome pathway. In Arabidopsis, clock related MYB proteins RVE4/RVE8 plays an direct transcriptional activators of DREB1 expression in cold stress. In unstressed condition CCA1 and LHY suppressed DREB1 expression, wheras in stressed condition RVE4/RVE8 translocate from cytoplasm to the nucleus and induces the expression of CBFs/DREB1 through cis acting element EE by rapidly degrading CCA1and LHY. Abbreviations: RLK, receptors like protein kinases; LRR-RLK leucine-rich repeats receptor-like protein kinase; CNGC, cyclic nucleotide-gated calcium channel; GLRs, glutamate-like receptor homologs; Ca^2+^, calcium ion; calcium binding proteins like CaM, calmodulin; CML, CaM-like proteins; CDPKs, Ca^2+^ dependent protein kinases; CBLs, calcineurin B-like proteins; MAPK, mitogen-activated protein kinase; CBFs, C-repeat Binding Factors; ICE, Inducer of CBF Expression; COR, cold-responsive; ROS, reactive oxygen species; NO, nitric oxide; CRPK1, cold-responsive protein kinase 1; RVE4/8, reveille4/8; lhy-cca1-Like1 (/LCL1); CCA1, circadian clock’s oscillator component circadian clock-associated1; LHY, late elongated hypocotyl; EE, cis acting element; TFs, transcription factors; DREB1, dehydration responsive element binding-protein 1.

The early events upon cold stress include changes in cell membrane structure and lipid composition that provide the basis of low temperature sensing (Pareek et al., 2017; Guo et al., 2018; Poovaiah and Du, 2018; Guan et al., 2021). These changes induce a downstream cold signaling cascade by changing the ion and metabolite transport and redox state of the cell (Steponkus, 1984; Carpaneto et al., 2007; Jeon & Kim, 2013; Zhang et al., 2021a). Many plasma-membrane localized receptors, such as receptors like protein kinases (RLK) and leucine-rich repeats receptor-like protein kinase (LRR-RLK) have been shown to induce cold signaling pathways (Chen et al., 2021; Ye et al., 201; Su. et al., 2022). In addition, ion leakage, which is a common symptom of cold stress in plants, also causes Ca^2+^ changes when temperature ebbs. Perception of cold stress on the plasma membrane activates Ca^2+^ permeable channel that leads to the release of Ca^2+^ inside the cell. However, the frequency, duration, and amplitude of calcium ions, combinedly known as calcium signature, depends on the strength of stress condition (Zheng et al., 2021). Ca^2+^ imaging based on aequorin and yellow Cameleon has provided evidence of transient cold-induced Ca^2+^ channel activation in Arabidopsis (Krebs et al., 2012; Sun et al., 2021). Further experiments with Arabidopsis and moss show that cyclic nucleotide-gated calcium channel (CNGC) and glutamate-like receptor homologs (GLRs) function in Ca^2+^ signaling (Finka et al., 2012; Wang J. et. al., 2021; Ghosh et al., 2022). These Ca^2+^ are sensed by many calcium binding proteins like calmodulin (CaM), CaM-like proteins (CML), Ca^2+^ dependent protein kinases (CDPKs), and calcineurin B-like proteins (CBLs), which are essential for the regulation of CBF/COR gene expression (Huang et al., 2011; Zeng et al., 2015; Atif et al., 2019; Zhang et al., 2022). Also, calcium-binding proteins induce cold tolerance by modulating different mitogen-activated protein kinase (MAPK) through phosphorylation at their threonine and tyrosine residue that finally interacts with ICE1 and controls the expression of CBF genes (Yuan et al., 2018; Figure 1).

Besides Ca^2+^ and reactive oxygen species (ROS), nitric oxide (NO) is also a prominent component of a cold signaling pathway in plants (Figure 1). ROS is a highly reactive, short-lived secondary messenger and has a conserved signaling pathway in diverse stress conditions (Choudhury et al., 2013; You and Chan, 2015; Lim et al., 2019). Production of ROS (superoxide, hydroxyl radicals, and hydrogen peroxide) upon stress encounter is one of the early steps, and if its accumulation exceeds the threshold level, it could harm to lipid, protein, RNA, and DNA and generate oxidative stress in the cell (Mittler, 2002; Sharma et al., 2021). The ROS enters the cell through aquaporin membrane proteins, perceived by membrane receptors and modifies the cytoplasmic proteins to regulate signaling and cellular processes. For example, mucolipin 1 (MCOLN1; a member of the transient receptor potential channel gene family) is a ROS sensor in lysosomes that regulates autophagy, hydrogen peroxide sensor 1 (HPC1; LRR receptor kinase gene family) and GUARD CELL HYDROGEN PEROXIDE-RESISTANT1 (GHR1; plasma membrane LRR receptor kinase gene family) is a H_2_O_2_ sensor in Arabidopsis regulate Ca^2+^ driven stomatal movement (Hua et al., 2012; Zhang et al., 2016; Wu et al., 2020). Previous reports provide evidence that Ca^2+^ and ROS are interlinked and low ROS level induces Ca^2+^ influx into the cytoplasm (Mazars et al., 2010; Verhage, 2021). The increased Ca^2+^ activates the NADPH to generate more ROS, which is then converted to H_2_O_2_ under the influence of superoxide dismutase. Thus, ROS and Ca^2+^ regulate each other’s concentration, and this cross-talk controls the expression of defense gene in the nucleus (Mazars et al., 2010). The trade-off between ROS and Ca^2+^ in response to cold stress is a matter of debate and needs to dissect their role in cold tolerance. NO is another crucial signaling molecule, and its role in combating abiotic stress has also been studied well (Shi et al., 2012; Puyaubert and Baudouin, 2014; Fancy et al., 2017; Liu et al., 2021). NO has also been found to be working in association with hormones such as ABA, jasmonic acid, ethylene, and other molecules like Ca^2+^, phosphatidic acid, H_2_O_2,_ and melatonin to mitigate cold stress (Costa-Broseta et al., 2018; Feng et al., 2021). Therefore, it is prudent to assume that NO should play a crucial role in combating cold stress. The previous study has shown that level of endogenous NO increases with cold acclimation due to enhanced activity of the *NITRATE REDUCTASE 1* gene (Zhao et al., 2009). Further, trehalose also triggers NO upon cold stress (Liu et al., 2021). It also acts as an intermediate in lipid-based signaling and gene regulation during cold acclimation (Cantrel et al., 2011; Sohag et al., 2020). Triple mutants *nia1nia2noa1-2* is impaired in the nitrate reductase (NIA/NR) and Nitric Oxide-Associated (NOA1)-mediated NO production, and are thus NO deficient. Study in Arabidopsis has shed light on the importance of NO-induced cold acclimation. In this study, the author demonstrated that NO accumulation is essential for cold acclimation response through CBF-dependent and CBF-independent gene expression (Costa-Broseta et al., 2019).

## C-repeat binding factors and cold-responsive signaling pathway

Once the temperature goes below the optimum, the *COR* gene springs into action to maintain homeostasis by mitigating the impact of cold stress. The first type of COR gene-dependent response involves encoding proteins required for the biosynthesis of osmoprotectants, cryoprotectants, protein kinases, lipid, hormone, and stress-responsive proteins like AFPs, HSPs, LEA, dehydrins that are directly involved in cold tolerance (Holmberge and Bülow, 1998; Thomashow, 2010; Kidokoro et al., 2022). Other sets of COR gene-dependent responses include genes such as early response to dehydration, low temperature-induced, response to abscisic acid, and cold-Induced transcription factors (Alves et al., 2011; Shi et al., 2015; Cao et al., 2021). The expression of COR genes is regulated by both CBF-dependent and CBF-independent pathways. CBFs are the transcription factor belonging to the superfamily ethylene-responsive element-binding factors and *APETALA2* (AP2/ERF), which recognize RCCGAC, a c-repeat dehydration-responsive element (CRT/DRE) (Akhtar et al., 2012; Zhu et al., 2021). CBFs have signature sequences, PKK/RPAGRxKFxETRHP and DSAWR, distinguishing them from other superfamily members (Canella et al., 2010; Fan et al., 2021). Out of four CBF genes found in the Arabidopsis genome, three *CBF* genes (*CBF1*, *CBF2*, *CBF3*) are induced by cold, whereas *CBF4* is induced by drought and salt stress. *CBF1*, *CBF2*, and *CBF3* are also known as DREB1b, DREB1c, and DREB1a, respectively. Of the three cold-induced *CBF* genes, only *CBF1* and *CBF3* positively regulate cold acclimation, whereas *CBF2* negatively regulates both *CBF 1* and *CBF3* by inducing different sets of genes (Novillo et al., 2007). A recent study suggests that CBF is also under the regulation of redox-dependent structural changes by Thioredoxin-H2(Trx-h2), a cytosolic redox protein, which enhances its function in the cold stress mitigation pathway. Trx-h2, which resides in the cytoplasm under normal conditions, upon cold stress migrates to the nucleus and binds to CBF and reduces the oxidized/inactive CBF (Lee et al., 2021). In CBF-dependent COR regulation, C-repeat binding factor/dehydration responsive element binding-protein-cold regulated (ICE-CBF3/DREB1-COR) is the major pathway regulating cold stress. CBF is under the direct control of several positive and negative regulatory elements and chromatin remodeling complexes (Ding et al., 2019). A recent study by Kidokoro et al. (2021) reports the circadian clock’s oscillator component circadian clock-associated1 (CCA1) and late elongated hypocotyl (LHY) negatively regulate DREB1 expression under normal growth conditions. Upon cold stress, CCA1 and LHY were rapidly degraded, followed by translocation of MYB transcription factor reveille4/lhy-cca1-Like1 (RVE4/LCL1) and RVE8/LCL5 from the cytoplasm to the nucleus and inducing the expression of DREB1 through cis-acting element EE (Kidokoro et al., 2021).

Different hormones also known to regulate CBFs. For example, gibberellic acid (GA) metabolism and signaling are under the target of cold stress. Cold induction leads to the activation of Gibberellin 2-oxidase (GA2ox), which leads to the hydroxylation and inactivation of bioactive GA. Moreover, overexpression of CBF leads to enhanced DELLA protein accumulation through post-translational modification (Eremina et al., 2016; Devireddy et al., 2021). The previous study has shown that GA deficient mutants of Arabidopsis and rice have altered chilling and freezing tolerance (Eremina et al., 2016). GA also controls CBF expression by mediating the regulation of PHYTOCHROME-INTERACTING FACTOR 4 (PIF4). In long day (LD; 16 h photoperiod) plants, PIF4 represses the expression of CBF and is directly controlled by DELLA protein. Further, jasmonic acid is the central hub of the JAZ-BBX37-ICE1-CBF pathway, which positively regulates cold stress tolerance (An et al., 2021). CBFs also associated with ABA metabolism genes such as 9-CIS-EPOXYCAROTENOID DIOXYGENASE 2 (NCED2), NCED3, NCED5, and cytochrome P450 monooxygenase (CYP707A3 and CYP707A4) (Song et al., 2021). The recent results from various studies suggest that CBF regulation is more abstruse than it appears. Evidence suggests that repression of CBF3/DREB1a in ice1-1 is achieved not only by genetic regulation but also by DNA methylation-mediated gene silencing and overexpression of ICE1 has no impact on cold-induced CBF3/DREB1a (Kidokoro et al., 2021). Mieura et al. have demonstrated that overexpression of ICE1 leads to increased expression of CBF1 and CBF3 by 30% and CBF2 by 24% (Miura et al., 2007). Moreover, the same group has also shown that ICE1 mutation (S403A) leads to stabilization of ICE1 and a twofold increase in the expression of CBF3/DREB1a during cold stress. Earlier studies have pointed out that ICE is the master regulator of cold-induced genes (Chinnusamy et al., 2003; Miura et al., 2007; Miura et al., 2011), however, recent study contradicts this hypothesis.

## Epigenetic regulation of cold stress

**FIGURE 2:**
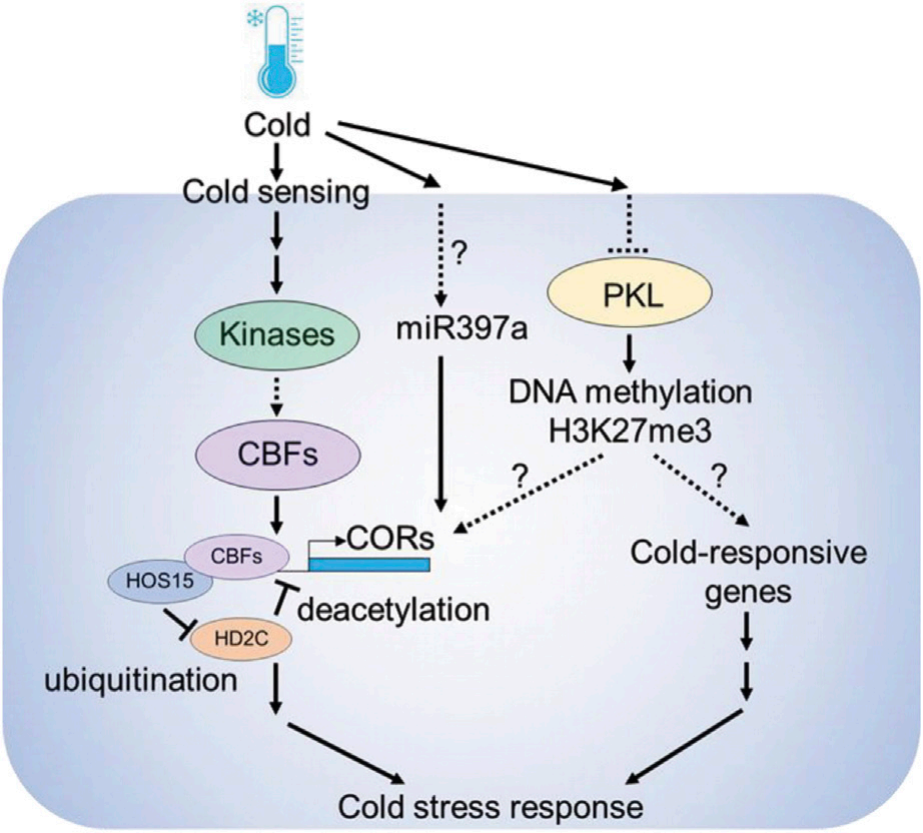
Epigenetic components involved in cold stress response in plants. Cold is sensed by upstream sensors followed by the activation of downstream gene expression. Under normal temperature conditions, HIGH EXPRESSION OF OSMOTICALLY RESPONSIVE GENES 15 (HOS15) interacts with HISTONE DEACETYLASE 2C (HD2C), and represses COLD RESPONSIVE (*COR*) gene expression by deacetylation. However, under cold stress conditions, HOS15 promotes HD2C degradation by ubiquitination, resulting in the increase of H3 acetylation on COR promoters. HOS15 also recruits CBFs to the COR promoters to activate COR gene expression. The chromatin remodeler PICKLE (PKL) modulates the chromatin status of *COR* genes through H3K27me3-dependent silencing. Also, under cold miR397a leads to the up-regulation of *COR* genes and enhanced cold tolerance.

Several epigenetic components including microRNAs, DNA methylation and histone modifications are involved in cold stress responses in plants (Figure 2). MicroRNAs (miRNAs) are known to play a role in cold-stress responses in plants (Megha et al., 2018). Analyzing small RNA libraries identified cold regulated miRNAs in Arabidopsis (Sunkar and Zhu, 2004). Furthermore, the Arabidopsis overexpression lines of miR397a showed higher CBFs and COR genes expression and improved tolerance against cold stress (Dong and Pei, 2014). SICKLE (SIC) is a proline-rich protein known to participate in miRNAs biogenesis, and *sic-1* mutant shows high sensitivity to cold and salt stress suggesting that microRNAs play a central role in stress responses (Zhan et al., 2012). Other than microRNAs, long noncoding RNAs are also important. Recently a cold-induced long noncoding RNA called SVALKA was identified, which negatively regulates CBF1 expression by producing a cryptic antisense transcript (Kindgren et al., 2018)

DNA methylation is a conserved epigenetic mark playing a crucial role in plant development and stress responses via modulating chromatin packaging and gene expression (Banerjee et al., 2017). Exposure to chilling and freezing stress resulted in the alterations of cytosine methylation in the alpine plant *Chorispora bungeana* as revealed by methylation-sensitive amplified fragment-length polymorphism (Song et al., 2015).

The chromatin remodeler PICKLE (PKL) plays a crucial role in cold stress responses. Arabidopsis *pkl* mutants are sensitive to cold stress and the expression of *CBF3* and *COR* family genes such as *COR15B* and *RD29A* were downregulated in *pkl* mutants (Yang et al., 2019). Also, PKL cooperates with the members of SWR1 chromatin remodeling complex members such as PHOTOPERIOD INDEPENDENT EARLY FLOWERING 1 (PIE1) to deposit H3K27me3 at gene loci (Carter et al., 2018). In Arabidopsis, cold stress caused the decline of H3K27me3 at COR15A and GALACTINOL SYNTHASE 3 (GOLS3) genes, suggesting that PKL modulates cold stress responses *via* H3K27me3 histone modifications at *COR* genes (Kwon et al., 2009). Also, enrichment in histone acetylation marks occurs in the promoters of several *COR* genes, including *COR15A* and *COR47* under cold stress (Zhu et al., 2008; Pavangadkar et al., 2010; Park et al., 2018). The process of histone acetylation is regulated by the concurrent action of HISTONE ACETYLTRANSFERASES (HATs) and HISTONE DEACETYLASES (HDACs). The Arabidopsis lines overexpressing HISTONE DEACETYLASE 2D (HD2D) were tolerant to cold stress as revealed by lesser accumulation of malondialdehyde (MDA) levels in transgenic plants (Han et al., 2016).

*HIGH EXPRESSION OF OSMOTICALLY RESPONSIVE GENE 1* (HOS1) is an E3 ubiquitin ligase that encodes a WD40-repeat protein. HOS1 is involved in the regulation of cold-responsive gene regulation by histone deacetylation (Zhu et al., 2008). HOS15 interacts with *HISTONE DEACETYLASE 2C* (HD2C) to regulate the expression of *COR* genes by binding to their promoters (Park et al., 2018). Under optimal temperature the HOS15-HD2C complex occupies the promoters of *COR* genes and induces the hypoacetylation of COR chromatin, leading to the inhibition of COR gene expression. However, HOS15 functions as an E3 ubiquitin ligase during cold stress by recruiting CUL4 (CULLIN4) to degrade HD2C. This process results in the hyperacetylation of H3 on COR chromatin, which consequently enhances the ability of CBFs to bind to COR promoters (Yang et al., 2019). Recently, it has been shown that, POWERDRESS (PWR) interacts with HOS15 to modulate the cold stress response. Arabidopsis *pwr* mutants show low expression of COR genes and are sensitive to freezing stress, suggesting that PWR-HOS15-HD2C histone-modifying complex regulates the COR gene expression and freezing tolerance in plants (Lim et al., 2020). These findings suggest that epigenetic regulation is an important mechanism for plant responses to cold stress.

The fluctuating and recurring exposure to low-temperature stress can result in cold stress memory, which can significantly improve plant fitness under cold stress conditions (Markovskaya et al., 2008). For instance, sustained cold stress exposure of *Arabidopsis thaliana* seedlings resulted in freezing tolerance which was further increased in response to triggering stress after 3 days of priming (Leuendorf et al., 2020). Importantly, the *cbf* mutants did not show much difference suggesting that CBFs have a limited function in cold stress priming. In the other study, *Arabidopsis* seedlings that were cold primed at 4°C, then placed at 20°C as a lag phase, and finally subjected to 4°C (cold stress) exhibited significant freezing tolerance. The results show that this is due to raffinose accumulation after the lag phase, suggesting that raffinose metabolism may be involved in the retention of cold memory (Zuther et al., 2019). Arabidopsis *pkl-1* mutant was less primable to cold stress than wild type as mutant showed poorer survival after being primed by mild cold stress. These findings suggest that chromatin remodeler PKL plays a major role in cold-stress memory (Yang et al., 2019). Vernalization is a well studied process that requires cold, regulated epigenetically by *POLYCOMB REPRESSIVE COMPLEX 2* (*PRC2*) to mediate the suppression of a flower repressor *FLOWERING LOCUS C* (*FLC*) (Schubert et al., 2006). Overall, epigenetic components are involved in regulating cold induced stress and developmental responses in plants.

## Cold adaptation in plants

Adaptation is a long term evolutionary process, whereas acclimation is a short term process contributing to overcome stress episodes. Both these processes contribute to combat the environmental odds and ensure plant survival. Cold acclimation occurs when temperature goes below normal at which plant is adapted to complete its life cycle.

### Morphological adaptation

**FIGURE 3:**
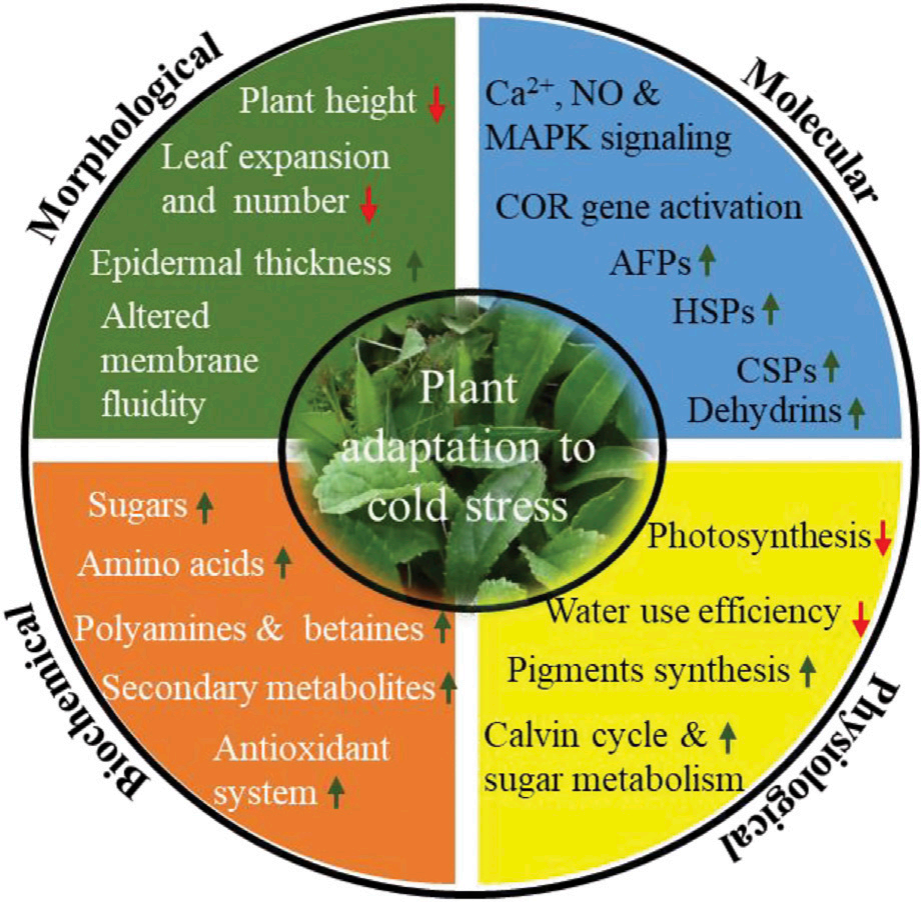
Different morphological, biochemical, physiological and molecular mitigation strategy acquired by plants upon cold stress. Change in different mitigation strategy are indicated by arrow (up) indicates increased concentration/expression, whereas arrow (down) indicates decreased concentration/expression). Abbreviations: Ca^2+^, calcium ion; NO, nitric oxide; MAPK, mitogen-activated protein kinase; *COR* genes, cold responsive genes; AFPs, antifreeze proteins; HSPs, heat shock proteins; CSPs, cold shock proteins.

At the morphological level, plants reduce their height, leaf show reduced expansion and numbers, increase epidermal thickens, and induce rigidification of plasma membrane by changing the nature and composition of membrane lipids (Figure 3). Under low-temperature conditions, cells get dehydrated, which results in osmotic stress that impacts on membrane integrity and permeability. Parallelly cold/freezing condition causes metabolic disbalance in the cytomembrane, resulting in excessive accumulation of ROS that leads to oxidative stress. Consequently, the cell membranes get damaged. Therefore, the plasma membrane maintains its structural and functional integrity by increasing lipid unsaturation, altering lipid class/composition and lipid/protein ratio (Takahashi et al., 2013; De, 2014; Holthuis and Menon, 2014; Wang B. et al., 2020). Membrane fluidity is largely determined by the desaturation of fatty acids and fractions of phospholipids, galactolipids, sphingolipids, and sterols. It has been reported earlier that many plants, including monocotyledonous, dicotyledonous, herbaceous, and woody like oat, rye, mulberry, orchard grass, and Arabidopsis have increased phospholipid (phosphatidylcholine and phosphatidylethanolamine) and decreased sterols and cerebrosides composition of the plasma membrane in response to cold stress (Uemura and Steponkus, 1999; Uemura et al., 2006). In contrast, Zhao et al. (2021) reported approximately 10% decrease in phosphatidylcholine in maize under low temperature suggesting regulation of membrane lipid composition predominantly takes place to counter low-temperature stress (Zhao et al., 2021). In addition, many lipid pathway enzymes get expressed to increase the proportion of unsaturated fatty acids. For example, genetic studies suggest that fatty acid desaturase (FAD) likely induces cold tolerance in *tetrahymena* thermophile to regulate membrane fluidity (Granel et al., 2019).

### Biochemical adaptation

Further, to mitigate these effects at biochemical level, plants synthesize and accumulate an array of cryoprotectant *viz.* soluble sugars, specific amino acids (Proline, glycine, alanine and serine), polyamines, betaines and secondary metabolites (Ramazan et al., 2022; Saddhe et al., 2021; Ming et al., 2021; Figure 2). Sugars like sucrose, glucose, fructose, galactose, raffinose, and trehalose regulate osmotic potential, prevent ice crystal formation, scavenges ROS, and thus increases membrane stability in order to survive in cold stress. In addition, sugars also act as signaling molecule involved in plant growth and development as well as in stress conditions. A large number of articles have been reported that describe the possible link between sugars accumulation and cold tolerance in plants (Heidarvand and Amiri, 2010; Tarkowski and Van den Ende, 2015). Proline is considered a biomarker for cold tolerance and has been accumulated in cold adapted plants like *A. thaliana*, Chickpea, barley, and winter wheat (Kumar and Yadav, 2009; Primo-Capella et al., 2021). Proline maintains osmotic potential, stabilize membrane and proteins, scavenge ROS, and regulates osmotic stress-related gene expression. Further, polyamines (PAs), particularly putrescine, spermidine, and spermine accumulation and degree of abiotic stress tolerance have been discussed in many plants (Gill & Tuteja, 2010; Tiburcio et al., 2014; Alcázar et al., 2020). In cold/freezing stress, PAs increases osmolyte accumulation, control redox homeostasis, stabilize membranes, promote seed germination, improve fruit quality, protect photosynthetic apparatus and regulate gene expression (Oufir et al., 2008; Alcázar et al., 2020). Excessive accumulation of ROS species warrants a greater concentration of antioxidants to neutralize the harmful impact of ROS. It leads to the synthesis of secondary metabolites including phenols, flavonoids, coumarins, catechins, tocopherols, cinnamic acid derivatives, lignins, and polyfunctional organic acids (Ahmed et al., 2015; Robe et al., 2021; Shkryl et al., 2021).

### Physiological adaptation

At the physiological level, photosynthesis is strongly affected by cold/freezing stress. Cold/freezing stress damage chloroplast membrane integrity, decreases the efficiency of photosystems I and II, intercellular CO_2_, chlorophyll a, and chlorophyll b, which in turn reduces net photosynthesis (Lin et al., 2007; Gao et al., 2022; Figure 3). In order to maintain photosynthetic efficiency, plant synthesize accessory pigments like carotenoids, violaxanthin, antheraxanthin, and zeaxanthin, increases the activity of enzymes associated with the calvin cycle and sugar metabolism as well as increases the synthesis of D1 protein, which is essential to maintain the efficiency of PSII (Fang et al., 2019; Lu K. et. al., 2021; Wu et al., 2021). Cold stress also alters the morphology and anatomy of stomata. Results from stevia and various experiments with other plants has sown that stomatal size increases under cold stress (Hajihashemi et al., 2018). Altered stomatal morphology might cause a significant reduction in the intracellular CO_2_ concentration and water use efficiency (https://doi.org/10.3389/fpls.2018.01430, 2018).

### Molecular adaptation

At the molecular level, plants synthesized stress-responsive protein like AFPss, HSPs, CSPs, LEA and dehydrins to assist protein folding and refolding activities, preventing the denaturation of globular macromolecule and cellular protein transport and inhibition of ice crystal growth (Figure 3). AFPs are the most important proteins, which we discuss in the next section.

## Antifreeze proteins and their role in cold tolerance

To survive in freezing stress, cold-hardy plants produce a specific type of protein called AFPs, which lowers the freezing temperature of the cytosol, inhibits the growth of ice-crystal in the apoplast, and attenuates freezing damage. AFPs were first discovered in arctic fish when De Vries and Wohlschlag discovered some plasma protein with the capacity to lower the freezing point of blood (DeVries and Wohlschlag, 1969). Few were proteinaceous and termed as AFPs, and others were glycosylated, named as antifreeze glycoproteins (AFGPs). These proteins are combinedly known as AF(G)Ps. Since its first discovery in marine teleosts, AF(G)Ps now have been reported from plants (Griffith et al., 1992a), molds fungi (Hoshino et al., 2003), sea-ice diatoms (Gwak et al., 2010), snow algae (Leya, 2013), and bacteria (Singh et al., 2014). The evolution of AF(G)Ps genes is thought to be done under adaptive conflict/environmental pressure by intra-gene/whole gene duplication, sequence divergence of selected genes like C-type lectin, trypsinogen, and sialic acid synthase, and from non-coding DNA sequences (Chen et al., 1997; Deng et al., 2010; Sorhannus, 2012; Zhuang et al., 2019). Although both AFPs and AFGPs have similar functions to mitigate freezing stress, they are different structurally. AFPs have distinct primary, secondary and tertiary structures, whereas AFGPs have repeated tripeptide units Ala-Ala-Thr (Harding et al., 2003; Urbańczyk et al., 2017; Sun et al., 2021). AFPs act in a non-colligative manner, and their unique structure equips them to bind to the minute ice-crystals and prevent their growth (Chapsky and Rubinsky, 1997; Carvajal-Rondanelli et al., 2011). Thermal hysteresis (TH) and ice recrystallization inhibition (IRI) are the two distinct properties of AFPs. TH refers to the separation of melting and freezing point, whereas IRI is the ability to inhibit the growth of ice crystals. Few reports have discussed the properties of AFPs in detail (Kuiper et al., 2001; Zhang et al., 2004; Middleton et al., 2009; Gupta and Deswal, 2014; Maddah et al., 2021). Here, in this review, we will majorly focus on plant AFPs in brief.

### Plant antifreeze proteins

In plants, AFPs were discovered for the first time in winter rye (Griffith et al., 1992a). It was demonstrated that the apoplastic extract from rye leaves acclimated for cold has a similar capacity to modify the ice formation as seen in fish and insects. These apoplastic extracts changed the ice-crystal morphology and had thermal hysteresis characteristics unique to the AFPs found in the animal kingdom. Since then, AFPs have been reported from more than 60 plants, including monocots, dicots, and gymnosperms (Smallwood et al., 1999; Wisniewski et al., 1999; Kuiper et al., 2001; Kawahara et al., 2009; Lauresen et al., 2011; Gupta and Deswal, 2012; Wang Q. et al., 2020; Arya et al., 2021; Yu et al., 2021). The AFPs from plants are different from the AFPs reported in animals in that it does not act as a cryoprotectant but as an ice interacting protein (Griffith et al., 2005). Plant AFPs, in general, were found to have low TH of around 0.1–0.5°C (Kuiper et al., 2001; Zhang et al., 2004) and high IRI compared to fishes, insects, and bacterial AFPs (2–13°C), however, plant AFPs with higher TH has also been reported (Gupta and Deswal, 2014). AFPs with low TH and high IRI seem to be of an evolutionary adaptation in plants as low TH allows the more controlled growth of ice crystals, and high IRI allows AFP to work even at minute concentrations.

### Ice binding sites of antifreeze proteins

AFPs bind to ice using ice-binding site, but to date, there is no consensus on the actual mechanism of this interaction. Studies have shown that amino acid sequence composition (Davies and Sykes, 1997), secondary structure like beta-strand rich proteins (Lu et al., 2002) motifs such as N-acetyl group at C-2 peptide chain containing O-glycosidic linkage (Urabńczyk et al., 2017), gamma -methyl group at threonine (Chakrabarty and Jana, 2019) and ice like motif (Hudait et al., 2018) assist the binding. Though ice-binding site is mainly hydrophobic, a recent molecular study has shown that hydrophobicity and hydrophilicity contribute to the ice-binding mechanism (Hudait et al., 2018). Recent work highlights the importance of hydration shell (the sphere of water molecule around each dissolved ion) in AFP and ice interactions through the use of FTIR spectroscopy and self-modelling curve resolution (Zanetti-Polzi et al., 2019). Another study has shown that higher water density at non-ice binding surfaces also contributes to AFP hyperactivity (Biswas et al., 2021). Diverse anchored clathrate motifs have also been found in assisting the ice binding activity of AFP (Hudait et al., 2018). The clathrate motif model states that AFP forms cages around the methyl group on the ice-binding surface by organizing surrounding water molecules into an ice-like lattice and thus forming a quasi-liquid like layer between water and then merging with the already formed ice-crystals. Taking the cues from fishes and insects AFPs, Walker et al. presented the theoretical model of Lolium perenne AFP (Kuiper et al., 2001). The predicted model has extended flat beta-sheet to its opposite side, similar to that of the beta helical structure of insect AFP. It also indicated the presence of two ice-binding sites. This duplication of the ice-binding site might explain the superior IRI of plant AFP. Later on, a site-directed mutation study by the same group showed that only one acts as an actual ice-binding site out of the two putative ice-binding sites. Their experimental study confirmed the theoretical model, which predicted the ice-binding site to be planar, hydrophobic, and of high order (Middleton et al., 2009). Unlike animal AFPs, which have been divided into four distinct groups, type I-IV (Xiang et al., 2020), plant AFPs have not been classified due to immense diversity (Bredow and Walker, 2017). Studies in this regard have shown that plant AFPs can be classified on the basis of amino acid composition, secondary structure, presence of certain motifs, and its homology with other proteins. For example, a 118 residues long, heat-stable, hydrophilic, AFP isolated from ryegrass was found to have repeating motifs of seven conserved residues XXNXVG and no homology with other AFP (Sidebottom et al., 2000). Similarly, DcAFP, isolated from carrot, shares sequence similarity with POLYGALACTURONASE INHIBITOR PROTEIN family from apoplastic having LEUCINE RICH REPEAT (Dang-Quan et al., 2006). Furthermore, Plakestrin Homology, WRKY proteins, pathogenesis-related proteins such as 3-beta-glucosidase and thaumatin-like proteins have also been identified in the plant as AFPs (Table 1).

**TABLE 1:** List of antifreez proteins reported in plants.

Plant origin	Protein name	Secondary structure composition	Domain similarity	Characteristics features	Localisation/References
*Secale cereale*	Glc AFP	Alpha helix-0%	Endoglucanase	O-linked glycosylation	Apoplastic/ Hon et al., 1995
		Extended strand- 48.28%			
		Coil- 51.72%			
	Cht AFP	Beta rich	Class I Endochitinase	Hexagonal bipyramidal structure of ice crystal	Secretory pathway/Hon et al., 1995; Yeh et al., 2000
	Cht AFP	Beta rich	Class II Endochitinase		
	TLP AFP	Beta rich	Thaumatin like domain		Phloem tissue/ Hon et al., 1995
*Daucus carota*	dcAFP	Alpha + Beta	Leucine rich repeat	N linked glycosylation	Secretory pathway/Ding et al., 2014
				High level IRI	
*Lolium perenne*	lpAFP	NA	NA	O linked glycosylation	Secretory pathway/Kuiper et al., 2001
*Ricinus communis*	rcAFP	Beta rich	Plant agglutinin	N and O linked glycosylation	NA/ Muthukumaran et al., 2011
	rcAFP	Beta rich	Plakesterin homology	NA	
*Chlorella vulgaris*	cvAFP	Alpha	NA	NA	Chloroplast
*Hippophae rhamnoids*	hr berry AFP	Alpha helix -41.03%	TLR and LRR	Hexagonal ice shaping	Cytoplasmic/Gupta and Deswal, 2012
		Beta sheet - 14.89%			
		Coil - 44%			
	hr berry AFP	Alpha helix -41.03%	TLR and LRR	Hexagonal ice shaping	Cytoplasmic/Gupta and Deswal, 2012
		Beta sheet - 14.89%			
		Coil - 44%			
	hr leaf AFP I	Alpha helix -28.82%	LRR	NA	Extracellular/Gupta and Deswal, 2012
		Beta sheet - 20.83%			
		Coil - 50.35%			
	Hr leaf II	Alpha helix -25.42%	Cystein rich secretory protein 5	NA	Cell wall/Gupta and Deswal, 2012
		Beta sheet - 9.69%			
		Coil - 64.89%			
*Solanum dulcumara*	STHP-64	Alpha helix- 18.78	WRKY	N and O limked glycosylation	Cytoplasmic/ Huang et al., 2002
		Extended strand- 19.80			
		Coil-61.42			
*Raphanus sativas*	rsAFP	NA	NA	Hexagonal ice shaping	Apoplastic/Wisniewski et al., 2020
*Triticum aestivum*	taAFP	Alpha helix - 13.14%	LRR	N and O linked Glycosylation	Secretory pathway/Zhang et al., 2007
		Extended strand - 25.14%		Heat stable AFP	
		Coil - 61.71%		High IRI	
*Picea abies*	paAFP	NA	Chitinase	No glycosylation	Apoplastic/Jarząbek et al., 2009
				Bipyramidal Ice crystals	
*Deschampsia antarctica*	daAFP	NA	LRR	O linked glycosylation	Secretory pathway/Cid et al., 2018
*Populus suaveolens*	psAFP	Alpha helix - 37.75%	Plakestrin homology	No glycosylation	NA/ Muthukumaran et al., 2011
		Extended strand - 19.87%			
		Coil - 42.38%			
*Festuca pratensis*	fpAFP	Alpha helix-NA	LRR	N and O-linked glycosylation	Chloroplast/ Cid et al., 2018; Muthukumaran et al., 2011
		Extended strand- 38.26%			
		Coil- 61.74			

### Extraction, purification, and identification of antifreeze proteins

Most of the research of AFPs has been centered on extraction, purification, and identification of AFPs, evaluation of antifreeze activity, and its implication for the development of cold resilient plants. To study AFPs, knowledge of AFP location is essential. In woody, herbaceous, and gramineous plant species, most AFP is localized in bark, root tissue, and leaf blades, respectively. For extraction of AFPs from bark/root tissue, conventional methods like grinding and stirring, while for leaf blades, the infiltration-centrifugation method is used (Chincinska, 2021).The infiltration-centrifugation method has gained recent attention because it collects apoplastic fluid without protoplasmic contamination. Once the antifreeze proteins are extracted, they are purified using conventional techniques like ultrafiltration, column chromatography, ion exchange, and ammonium precipitation, and a classical method like ice affinity chromatogram (Tasaki and Okada, 2008; Sharma et al., 2019) followed by mass spectrometry for identification. A newer method like falling water ice affinity purification has also been reported (Adar et al., 2018). This method takes advantage of the affinity of ice-binding proteins for ice. In this purification method, the crude hydrolysate falls on a chilled vertical surface of a commercial ice machine; ice-binding proteins binds to the ice, whereas non-ice-binding proteins do not bind to ice. Many plant species have been used for isolation, purification, and identification of AFPs, such as bittersweet nightshade, winter rye, carrot, ryegrass, malting barley, oat, *Hipphoae rhamnides*, *Brassica juncea*, and *Ammopiptanthus nanus* (Griffith et al., 1992b; Gupta and Deswal, 2012; Ding et al., 2014; Ding et al., 2015; Arya et al., 2021; Yu et al., 2021). Further, nanolitre osmometry, differential scanning calorimeter, sucrose sandwich splat assay, and capillary assays are used to detect antifreeze activity in the AFPs extract /purified AFPs based on how AFP changes the growth of ice crystals. The former two measure TH, whereas the latter two are used to measure IRI. Recently, a colorimetric assay based on the change in color of the freeze-labile AuNP (Gold Nanoparticle) solution has also been widely used in studying AFPs (Park et al., 2013; Mitchell et al., 2015). Proteins with AFP properties degrade the AuNP, thus changing the color of the solution, whereas non-AFP protein doesn’t change the color of the solution. Furthermore, different *in silico* tools like AFPredictor, AFP-Pred, TargetFreeze, iAFP-Ense, afpCOOL, AFP-CMBPred, and AFP-LSE are also being used for predicting and analysis of AFP based on different principles (Table 2).

**TABLE 2:** List of *in silico* tools used for predicting and analysis of AFP.

Softwares	Prediction basis	References	Web access
AFPredictor	Surface-based pattern detection algorithm	Doxey and McConkey, (2006)	Freely available on request from the authors
AFP-Pred	Random forest approach	Kandaswamy et al. (2011)	No web server
AFP-PSSM	Support vector machine and position specific scoring matrix profiles	Zhao et al. (2012)	http: //59.73.198.144/AFP_PSSM/
AFP-PseAAC	Concept of pseudo amino acid composition	Mondal and Pai, (2014)	http://www.csbio.sjtu.edu.cn/bioinf/PseAAC/
AFP-Ensemble	Random forest classifiers and ensemble method	Yang et al. (2015)	http://afp.weka.cc/afp
TargetFreeze	Combination of weights using sequence evolutionary information and pseudo amino acid composition	He et al. (2015)	http://csbio.njust.edu.cn/bioinf/TargetFreeze
iAFP-Ense	Ensemble classifier	Xiao et al. (2016)	http://www.jci-bioinfo.cn/iAFP-Ense
CryoProtect	Amino acid composition, dipeptide composition, and physicochemical property	Pratiwi et al. (2017)	http://codes.bio/cryoprotect/
AFP-LSE	Latent space learning of K-spaced amino acid pairs	Usman et al. (2020)	https://github.com/Shujaat123/AFP-LSE
AFP-CMBPred	Extending consensus sequence into multi-blocks evolutionary information	Ali et al. (2021)	--------

### Applications of antifreeze proteins

Based on AFPs properties to inhibit low temperature damage, the development of AFPs based products have found in various fields such as agriculture, industries, and medicine. In agriculture, it is used as a biofertilizer (Eskandri et al., 2020), germination promoter (Kyu et al., 2019), and to develop AFPs transgenic plants. In the past decade, several AFPs-transgenic plants have been developed to adapt to cold environments like maize (Zhang et al., 2021b), tomato (Balamurugan et al., 2018), tobacco (Huang et al., 2021), sweet potato (Lai et al., 2020). Such transgenic plants might provide humanity with food security in the near future in the wake of climate change. In the medicinal field, AFPs have found their uses in cryopreservation of organs, embryos, oocytes, and improving cryopreservation efficiency (Lee et al., 2015). The use of AFPs as a cryopreservent has an advantage compared to synthetic cryoprotectant agents like DMSO. It reduces the damage and mortality of the preserved organs, cells, and tissues. For example, AFPs from winter flounder have shown a better recovery rate of red blood cells post preservation (Stevens et al., 2022). A study by the group Tomas et al., has been demonstrated that the application of extracellular AFP enhances the protection of cell monolayers (Tomas et al., 2006). AFPs have also been used in minimally invasive surgery to destroy bad tissues (Liu et al., 2021) and improve the vitrification process of mouse oocytes (Robles et al., 2019). The global AFP market is expected to reach 26 million dollars by 2026 (https://www.marketsandmarkets.com/Market-Reports/antifreeze-protein-market-264931272.html). The major driving force in this increase is thought to be the frozen food industry. Studies have shown that the treatment of zachunni, cucumber (Hu et al., 2022), green beans (Kashyap et al., 2020), frozen desserts (Ma et al., 2022), vegetables (Kim et al., 2019), star fruit (Provesi et al., 2019), and beef (Hu et al., 2022) with AFP solution retains the original texture and sensory perception of frozen food post-preservation. Since artificial cryoprotectives like polyvenyl alcohol, polyampholyte, graphene oxide, cause health issues, natural AFPs can use as food preservatives without damaging health. In the material industry, AFPs have been utilized to make antifrosting, anti-icing polymers, and ceramics, which increase the safety measures in appliances (Eskandari et al., 2020).

## Engineering cold resilient plants and its application

The development of cold resilient plants is a demand for crops for the future. Historical methods such as conventional breeding to modern biotechnological techniques like genome editing such as clustered regularly interspaced short palindromic repeat (CRISPR) technology, epigenetic modification through altered methylation tagging, and other methods can be applied to develop chilling/freezing tolerant crops. Recent advancements in understanding the plant metabolic, transcriptomic, and signaling response to cold/freezing stress have aided and paved the path for the utilization of generated knowledge via different techniques. For example, QTL identified in several crops by genome-wide association studies with freezing tolerance can be used in breeding cold/freezing tolerant crops (Zhang et al., 2014; Huang et al., 2018; Wąsek et al., 2022). Although, there is limited success in developing cold resilient plants using traditional breeding approaches. The main reason behind this is that different responses in different plant species achieve cold tolerance, and mostly it is a combined effect of multiple factors at gene and metabolite levels. Thus, a multidisciplinary approach targeting multiple factors simultaneously can provide a better success rate in successfully implementing strategies to develop cold resilient crops using any method. Below are few strategies that can be applied to develop cold resilient crops.

### Strengthening the wall (primary defense)

Cold stress is received differently, and plants respond differently to them at various stages. Cell membrane systems are the primary site of freezing injury in plants, and making them more tolerant or insensitive toward freezing/cold stress can serve as the first line of defense in resisting cold. Increased cold stress tolerance has been achieved in several plants by either modulating cell membrane composition or manipulating genes and proteins involved in perceiving cold stress. For example, the level of membrane fatty acids and their saturation states is critical in determining low- temperature sensitivity. Increased unsaturation of membrane fatty acids leads to a decrease in low-temperature sensitivity. Thus, increasing the saturation of membrane fatty acids is an effective way to generate cold resilient plants (Zhang Y. et al., 2021). Similarly, Modulation of several other genes and proteins belonging to plasma membrane have shown a significant increase in cold/freezing tolerance in transgenic plants in tobacco (MpRCI, Feng et al., 2009), rice (OsSMP1, Zheng et al., 2021), Arabidopsis (PsCor413pm2, Zhou et al., 2018), (Feng et al., 2009; Zhou et al., 2018; Zheng et al., 2021).

### Primed/pre-activation of cold stress signaling

One of the best approaches to contest cold stress is to pre-activate the stress signaling response, which in turn activates the downstream pathway and prepares the plant to combat the stress. Cold stress is sensed and signalled through divergent but mostly conserved signaling cascades in different plant species with ICE-CBF-COR pathway. Pre/constitutive activation of this pathway genes has been and is one of the most effective and promising ways to develop cold resilient crops (Liu et al., 1998; Chinnusamy et al., 2003; Wang et al., 2008; Zhang H. et al., 2019). Overexpression of signaling genes helps in the preparedness of endogenous freeze tolerance machinery. Overexpression (both constitutive and inducible) of TFs primarily CBFs, has enhanced freezing tolerance in transgenic plants (Park and Chen 2006; Lv et al., 2020). Similar enhancement in freezing tolerance is also reported by overexpression of cold-induced DREB and ICE TFs (Wang et al., 2008; Zhang L. et al., 2019). In addition to TFs, overexpression of COR, and LEA genes, acting downstream of CBFs also enhances freezing tolerance in many plants (Artus et al., 1996; Hara et al., 2003; Ju et al., 2021).

### Increasing the warriors

Once a plant experience cold stress, it activates its warriors to defend and protect itself from the adverse effects. A plant cold stress warrior consists of different proteins and metabolites which protects plant cell from freezing. Sugars (trehalose, fructans), compatible solutes or osmolytes, proline, glycine betaine and many other small molecules and metabolites act like a warrior in scavenging ROS or providing osmoprotection. The concentration of these osmolytes and metabolites increases during cold stress; thus, increasing their amount in advance or under specific conditions is an efficient way to engineer plants for cold stress adaptation (Giri, 2011; Miranda et al., 2017). Sometimes a constitutive overexpression for the biosynthesis of these metabolites and solutes can interfere with the plant developmental process (Goddijn et al., 1997; Cortina and Culiáñez-Macià, 2005; Giri, 2011) while sometimes not (Jang et al., 2003: Su et al., 2006). Thus a better way is to express them under stress-inducible promoters (Su and Wu, 2004; Miranda et al., 2007; Iordachescu and Imai, 2008).

### Antifreeze proteins –new avenue to engineer cold resilient plants

Antifreeze proteins found mainly in cold region plants, microbes and animals, restricts formation of ice crystals within cell, and helps in the survival of plants in freezing environments. Recently, the function of AFPs and their role in providing plant freezing tolerance has gained importance. Several antifreeze proteins have been identified from many temperate plant species. Transferring single genes encoding antifreeze proteins to freezing-sensitive plants lowered their freezing temperatures by ∼1°C (Griffith and Yaish, 2004). Similarly, there are ice-binding proteins (IBP) present in microbes, animals and plants, which control the growth of ice crystals and mitigate freezing damage. Several of these proteins have been identified, and heterologous expression of these proteins in plants successfully lowered the freezing temperature (Hightower et al., 1991; Kenward et al., 1999; Khanna and Daggard, 2006). The use of plant IBP has proven to have more tolerance, and their overexpression led to decreased electrolyte leakage, indicating membrane protection and enhanced freeze survival at temperatures below –5°C (Fan et al., 2002; Zhang et al., 2010; Bredow et al., 2016). Recently, it has been demonstrated that the expression of more than one IBP isoform, as would be expressed endogenously in plants, further enhanced freeze survival in transgenic *A. thaliana* (Bredow et al., 2016).

In conclusion, cold/freezing tolerance is a multifaceted process regulated at different cellular levels, starting from the plasma membrane to intercellular processes involving genes and metabolites. Thus, the strategy to develop cold resilient plants depends on plant species, degree of resilience needed, technology applied, and selection of genes/metabolites. With advancements in the understanding of cold stress response, evolvement of new biotechnological tools for precise gene editing, and multiple gene-editing, the development of cold resilience plants is very well within reach.
